# Efficacy of Ion-Channel Inhibitors Amantadine, Memantine and Rimantadine for the Treatment of SARS-CoV-2 In Vitro

**DOI:** 10.3390/v13102082

**Published:** 2021-10-15

**Authors:** Yuyong Zhou, Karen A. Gammeltoft, Andrea Galli, Anna Offersgaard, Ulrik Fahnøe, Santseharay Ramirez, Jens Bukh, Judith M. Gottwein

**Affiliations:** 1 Copenhagen Hepatitis C Program (CO-HEP), Department of Infectious Diseases, Copenhagen University Hospital-Hvidovre, Kettegård Alle 30, 2650 Hvidovre, Denmark; yuyong.zhou@regionh.dk (Y.Z.); karen.anbro.gammeltoft@regionh.dk (K.A.G.); andrea.galli@regionh.dk (A.G.); anna.offersgaard@regionh.dk (A.O.); ulrik@sund.ku.dk (U.F.); santseharayra@sund.ku.dk (S.R.); jbukh@sund.ku.dk (J.B.); 2 Copenhagen Hepatitis C Program (CO-HEP), Department of Immunology and Microbiology, Faculty of Health and Medical Sciences, University of Copenhagen, Blegdamsvej 3B, 2200 Copenhagen, Denmark

**Keywords:** SARS-CoV-2, COVID-19, drug repurposing, ion-channel inhibitor, antiviral, hepatitis C virus p7 inhibitor, adamantane, remdesivir, combination treatment, barrier to escape

## Abstract

We report the in vitro efficacy of ion-channel inhibitors amantadine, memantine and rimantadine against severe acute respiratory syndrome coronavirus 2 (SARS-CoV-2). In VeroE6 cells, rimantadine was most potent followed by memantine and amantadine (50% effective concentrations: 36, 80 and 116 µM, respectively). Rimantadine also showed the highest selectivity index, followed by amantadine and memantine (17.3, 12.2 and 7.6, respectively). Similar results were observed in human hepatoma Huh7.5 and lung carcinoma A549-hACE2 cells. Inhibitors interacted in a similar antagonistic manner with remdesivir and had a similar barrier to viral escape. Rimantadine acted mainly at the viral post-entry level and partially at the viral entry level. Based on these results, rimantadine showed the most promise for treatment of SARS-CoV-2.

## 1. Introduction

The global coronavirus disease 2019 (COVID-19) pandemic caused by severe acute respiratory syndrome corona virus 2 (SARS-CoV-2) has resulted in >180 million infections and >4.4 million deaths (31 August 2021) [1,2]. The repurposing of approved drugs facilitates the definition of treatment regimens for new diseases in a cost- and time-effective manner. Remdesivir, originally developed for treatment of hepatitis C virus (HCV), acts by inhibiting the SARS-CoV-2 polymerase and is the only approved drug for the treatment of COVID-19 directly targeting a SARS-CoV-2 protein [3].

We investigated the effects of the adamantane derivatives amantadine, memantine and rimantadine (Figure S1) on SARS-CoV-2 in vitro. Amantadine and rimantadine inhibit viral ion-channels, such as the influenza A virus M2 and the HCV p7, were approved for the treatment of influenza A [4], and show activity against other viruses including beta-coronaviruses [5,6,7,8,9,10]. In addition, amantadine and memantine were approved for the treatment of neurodegenerative diseases [11], presumably blocking receptor-associated ion-channels [12]. We determined median effective concentrations (EC50) and median cytotoxic concentrations (CC50) in three physiologically relevant cell lines, including a human lung cell line. Further, we investigated interaction with remdesivir, barrier to viral escape and mode of action.

## 2. Materials and Methods

Further details are provided in Supplementary Materials.

### 2.1. Virus

SARS-CoV-2/human/Denmark/DK-AHH1/2020 was isolated from a Danish patient [13].

### 2.2. Inhibitors

Inhibitors were purchased (Sigma, Saint Louis, MO, USA), dissolved in DMSO (Sigma, Saint Louis, MO, USA) and stored at −20 °C.

### 2.3. Cells

African green monkey kidney VeroE6 cells, human hepatoma Huh7.5 cells and human lung carcinoma A549-hACE2 cells were cultured as described [13,14,15].

### 2.4. Longer-Term Treatments

SARS-CoV-2 infected VeroE6 cultures were treated immediately after virus inoculation and upon cell splitting [13,14,15]. Cultures were evaluated by immunostaining for SARS-CoV-2 spike protein upon cell splitting.

### 2.5. Next Generation Sequencing (NGS) of SARS-CoV-2 Genomes from Cell Culture Supernatant

SARS-CoV-2 RNA was extracted from cell culture supernatant with Trizol LS (Life Technologies, Carlsbad, CA, USA) and chloroform (Sigma, Saint Louis, MO, USA) and purified using RNA Clean and Concentrator™—5 (ZYMO, Irvine, CA, USA) [13]. Five overlapping amplicons were generated by reverse transcription polymerase chain reaction, library preparations were achieved using NEBNext Ultra II FS DNA library prep kit (New England BioLabs, Ipswich, MA, USA) and NGS analysis was performed as described [13].

### 2.6. Short-Term Concentration-Response Treatments

Single ion-channel inhibitor treatments were carried out in VeroE6, Huh7.5 and A549-hACE2 cells [13,14,15]. Remdesivir combination treatments were carried out in VeroE6 cells. VeroE6, Huh7.5 and A549-hACE2 cells in 96-well plates (Thermo Fischer Scientific, Roskilde, Denmark) were inoculated with SARS-CoV-2 at MOI 0.002, 0.02 or 0.003, respectively, and treated with inhibitor dilutions. MOI for these assays were selected based on virus titration assays carried out in the different cell lines as described [14], to obtain fully infected cultures whilst avoiding virus-induced cytopathic effects. Treatment conditions were tested in 7 replicates. Treatment plates included 14 infected-nontreated and 12 noninfected-nontreated wells. Cells were immunostained for SARS-CoV-2 spike protein after incubation for 46–50 h (VeroE6 and A549-hACE2 cells) or 70–74 h (Huh7.5 cells). Data points were given as residual infectivity (%) with standard errors of the means (SEM). Residual infectivity was determined as % SARS-CoV-2 spike positive cells in the infected-treated cultures relative to the means of the counts of positive cells in the infected-nontreated control cultures and was calculated as:

100× (count in infected-treated culture/mean of counts in infected-nontreated cultures).

### 2.7. Time-of-Addition Experiment with Rimantadine

VeroE6 cells in 96-well plates were inoculated with SARS-CoV-2 at MOI 0.01 with a 2-h infection phase and treated with 230 μM rimantadine at different timepoints post inoculation [15]. For entry treatment, rimantadine was added together with the virus at 0 h post inoculation and removed in the end of the 2-h viral infection phase. For post-entry treatment, rimantadine was added 2, 4 or 6 h post inoculation. Treatment conditions were tested in 6 replicates. Treatment plates included 12 infected-nontreated and 12 noninfected-nontreated wells. Cells were immunostained for SARS-CoV-2 spike protein after incubation for 46–50 h. Data points were given as % inhibition with SEM. % inhibition was determined as 100%–% residual infectivity (see previous section).

### 2.8. Immunostaining and Evaluation of 96-Well Plates for Short-Term Treatments and Time-of-Addition Experiment

Short-term treatment plates were stained with primary antibody SARS-CoV-2 spike chimeric monoclonal antibody (Sino Biological #40150-D004, Beijing, China) diluted 1:5000, secondary antibody F(ab’)2-goat anti-human IgG-Fc cross-adsorbed secondary antibody, HRP (Invitrogen#A24476, Carlsbad, CA, USA) or goat F(ab’)2 anti-human IgG–Fc (HRP), preadsorbed (Abcamab#98595, Cambridge, UK) diluted 1:2000, and DAB substrate BrightDAB kit (Immunologic#BS04-110, Duiven, Netherlands). Single SARS-CoV-2 spike protein positive cells were automatically counted using an ImmunoSpot series 5 UV Analyzer (CTL Europe GmbH, Bonn, Germany). Counts from infected-treated wells were related to the mean count of infected-nontreated wells to calculate the %residual infectivity for single inhibitor treatments and, in addition, the % inhibition for combination treatments and time-of-addition experiment [13,14,15].

### 2.9. Cell Viability Assays

Single ion-channel inhibitor treatments were evaluated in VeroE6, Huh7.5 and A549-hACE2 cells [13,14,15], while remdesivir combination treatments were evaluated in VeroE6 cells [14].

Cells in 96-well plates were treated with inhibitor dilutions. Treatment conditions were tested in 3–4 replicates. Treatment plates included 12 nontreated wells. Cell viability was evaluated using the CellTiter 96 aqueous one solution cell proliferation assay (Promega, Madison, WI, USA) after incubation for 46–50 h (VeroE6 and A549-hACE2 cells) or 70–74 h (Huh7.5 cells). Cell viability values of treated wells were related to the mean cell viability of nontreated wells to estimate % cell viability.

## 3. Results

### 3.1. Amantadine, Memantine and Rimantadine Showed Activity against SARS-CoV-2 In Vitro

**Figure 1:**
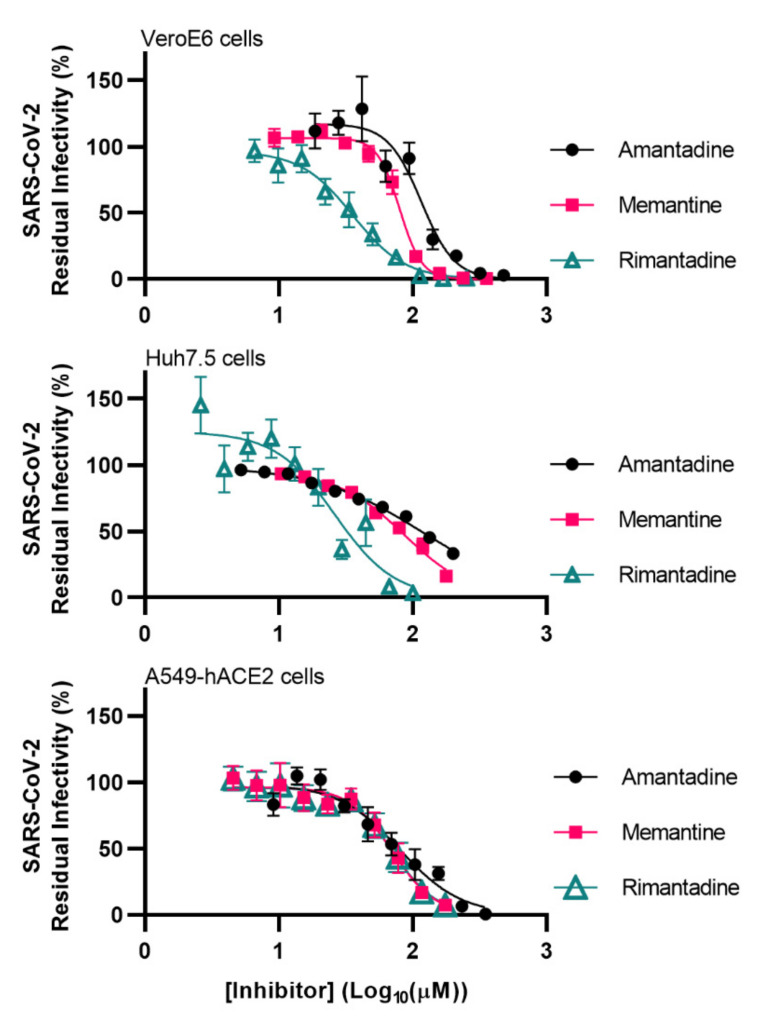
Potency of amantadine, memantine and rimantadine against SARS-CoV-2 in VeroE6 cells, Huh7.5 cells and A549-hACE2 cells. Short-term treatments of SARS-CoV-2 with the specified ion-channel inhibitors were carried out in the specified cell lines in 96-well plates. SARS-CoV-2 infected cells were visualized by immunostaining for spike protein and quantified by automated counting. Datapoints are means of counts from 7 replicate cultures ± SEM and represent % residual infectivity, determined as % SARS-CoV-2 positive cells relative to means of counts from infected-nontreated control cultures. Sigmoidal concentration-response curves were fitted and EC50 values were determined using Graphpad Prism 8.0.0 applying the formula Y = Top/(1 + 10^(Log10EC50-X)*HillSlope^). The tested inhibitor concentrations did not affect cell viability (Figures S2–S4).

To determine the potency of adamantane derivatives against SARS-CoV-2, we carried out 96-well based short-term concentration-response assays based on the quantification of infected cells by immunostaining for SARS-CoV-2 spike protein [13,14,15]. Assays were carried out in African green monkey VeroE6 cells, a prototype cell line for the evaluation of drug activity against SARS-CoV-2, human hepatoma Huh7.5 and human lung carcinoma A549-hACE2 cells. Used inhibitor concentrations did not result in a reduction of cell viability (relative cell viability > 90%), as shown in Figures S2–S4. Similar results were obtained in all cell types with EC50 values in the micromolar range. Rimantadine was most potent with EC50 of 36, 26 and 70 µM in VeroE6, Huh7.5 and A549-hACE2 cells, respectively. Memantine showed intermediate potency (EC50 of 80, 86, and 70 µM) and amantadine showed the lowest potency (EC50 of 116, 118, and 80 µM) (Figure 1, Table 1). At the highest used concentrations, all inhibitors had the capacity to fully inhibit SARS-CoV-2 in VeroE6 and A549-hACE2 cells, while slightly lower inhibition was achieved in Huh7.5 cells (Figure 1). Amantadine showed lower cytotoxicity than memantine and rimantadine (Table 1, Figures S2–S4). However, due to its comparatively high potency, rimantadine had the highest selectivity index (SI), while memantine had the lowest SI in all three cell lines (Table 1).

### 3.2. Amantadine, Memantine and Rimantadine Interacted in a Similar Antagonistic Manner with Remdesivir

**Figure 2:**
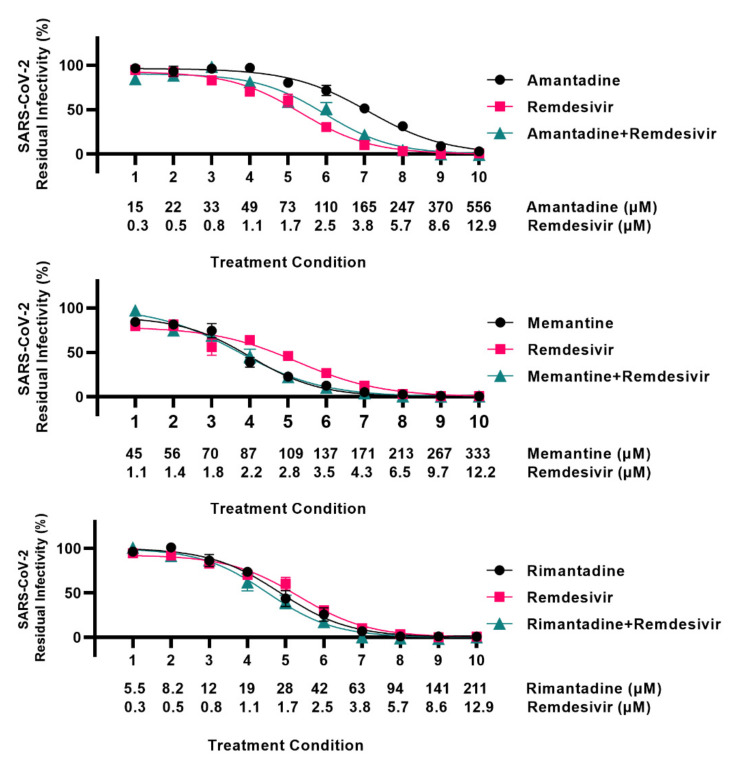
Analysis of interactions of adamantane derivatives with remdesivir in VeroE6 cells. VeroE6 cells seeded in 96-well plates were infected with SARS-CoV-2 and treated with serial dilutions of amantadine, memantine, rimantadine, or remdesivir, or a combination of these ion-channel inhibitors and remdesivir. SARS-CoV-2 infected cells were visualized by immunostaining for spike protein and quantified by automated counting. For each inhibitor pair to be evaluated, the treatment conditions indicated on the x-axis were applied. Each treatment condition was defined by a specific concentration of ion-channel inhibitor singly, a specific concentration of remdesivir singly, and a combination of these same concentrations of ion-channel inhibitor and remdesivir as indicated, resulting in three datapoints per treatment condition. Datapoints are means of counts from seven replicate cultures ± SEM and represent % residual infectivity, determined as % SARS-CoV-2 positive cells relative to means of counts from infected-nontreated control cultures. Sigmoidal concentration-response curves were fitted and EC50 values were determined using Graphpad Prism 8.0.0 applying the formula Y = Top/(1 + 10^(Log10EC50-X)*HillSlope^). The tested inhibitor concentrations did not affect cell viability (Figure S6).

To study interactions between ion-channel inhibitors and remdesivir, 96-well based combination treatments were carried out. SARS-CoV-2 infected VeroE6 cells were treated with ion-channel inhibitors singly or in combination with remdesivir, or with remdesivir alone. Inhibitor concentrations were selected based on previously determined EC50 values: for ion-channel inhibitors EC50 are given in Table 1, and for remdesivir EC50 was 2.5 µM, as previously reported [14]. For all three ion-channel inhibitors, the effect of the combination treatments did not exceed the effect of the single treatments (Figure 2, Supplementary Results, Table S1). Analysis using the method of Chou and Talalay [16] in the CompuSyn software [17] as described in Supplementary Methods revealed mostly antagonistic interactions between the ion-channel inhibitors and remdesivir (Supplementary Results, Figures S5 and S6 and Table S2).

### 3.3. Adamantane Derivatives Did Not Differ in Their Barrier to Viral Escape

**Figure 3:**
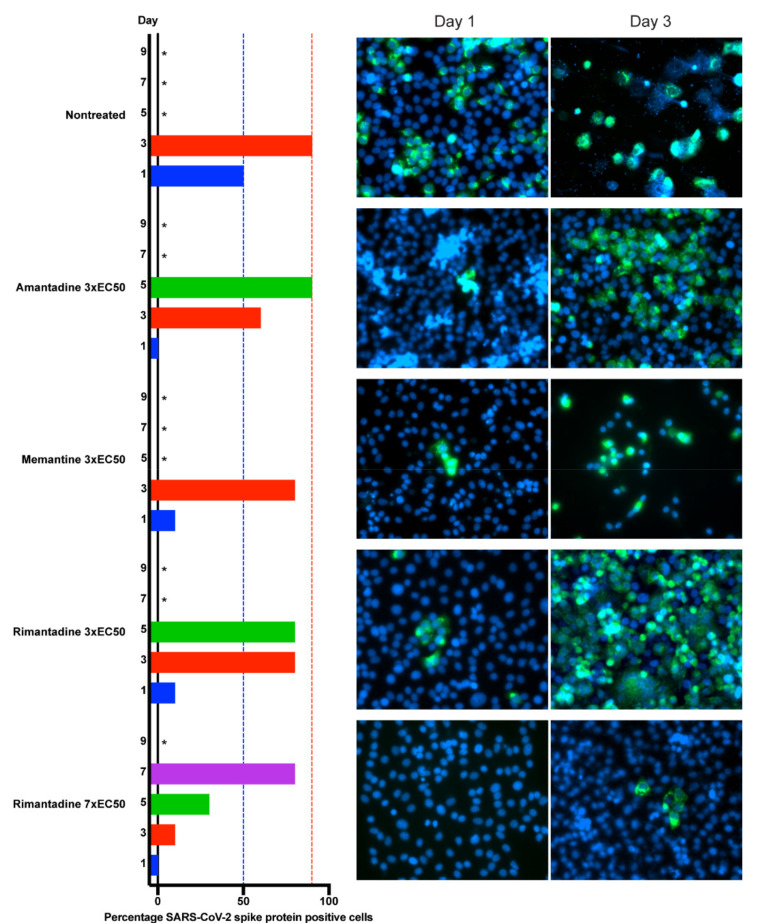
Comparison of barrier to SARS-CoV-2 escape of ion-channel inhibitors in VeroE6 cells. VeroE6 cells seeded in T25 cell culture flasks were infected with SARS-CoV-2 and treated with ion-channel inhibitors amantadine, memantine or rimantadine, at specified concentrations. Inhibitors were administered immediately after infection and on day 1, 3, 5 and 7 upon splitting of cells. % of SARS-CoV-2 infected cells on the specified days post infection was determined by immunostaining for SARS-CoV-2 spike protein (green) relative to counterstaining of cell nuclei with Hoechst dye (blue). Images were obtained using a Zeiss Axiovert microscope equipped with a 40× LD Plan-Neofluar objective. Cultures summarized in this figure are derived from different experimental setups, each including an infected-nontreated control culture, which showed viral spread comparable to that in the depicted representative culture. * Culture was terminated, or infection data not recorded, due to virus induced cell death.

To compare ion-channel inhibitors regarding their capacity to prevent SARS-CoV-2 spread under treatment, we carried out longer-term treatments of infected VeroE6 cells using amantadine, memantine and rimantadine at the highest possible equipotent concentrations (3-fold EC50), according to inhibitor cytotoxicities (Figure S2). Treatment with all inhibitors resulted in a similar delay of early viral spread kinetics on day 1 post infection and treatment initiation, while ≥80% of culture cells became infected on day 3–5, comparable to the nontreated control cultures (Figure 3). However, compared to the nontreated and the memantine treated cultures, somewhat reduced cytopathogenic effects were observed in the amantadine and rimantadine treated cultures. Thus, overall, the three inhibitors did not show major differences in their barrier to viral escape. The favorable SI of rimantadine enabled treatment with seven-fold EC50, resulting in additional viral suppression on days 3–5, while on day 7 ≥80% of culture cells became infected. To investigate whether the acquisition of substitutions might have facilitated viral escape, viruses from all cultures shown in Figure 3 derived at the peak of infection were subjected to NGS analysis. In memantine and rimantadine treated cultures, substitutions that were not found in the nontreated culture were detected, however, without apparent hotspots for substitutions (Table S3). Thus, inhibitors could only temporarily suppress SARS-CoV-2 at concentrations permissible in vitro according to inhibitor cytotoxicities.

### 3.4. Rimantadine Inhibited Infection with SARS-CoV-2 Mainly at the Viral Post-Entry Level

**Figure 4:**
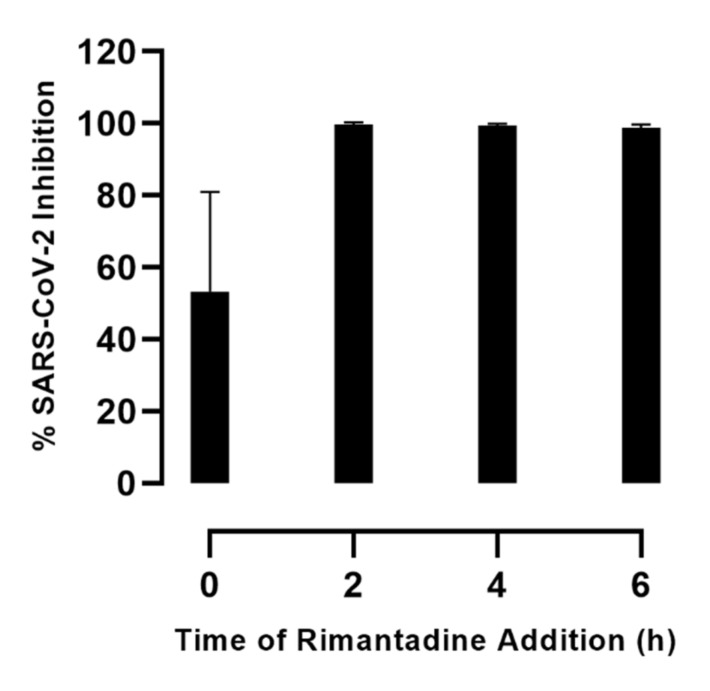
Time-of-rimantadine-addition assay in VeroE6 cells. VeroE6 cells seeded in 96-well plates were inoculated with SARS-CoV-2 during a 2-hour infection phase and treated with 230 µM rimantadine at different timepoints post viral inoculation. At 0 h, viral entry treatment; inhibitor was added together with the virus at 0 h post inoculation and was removed together with the virus following the 2-hour infection phase. At 2 h, 4 h or 6 h, post-entry treatment; inhibitor was added 2, 4 or 6 h post inoculation and maintained throughout the 48-hour incubation period. SARS-CoV-2 infected cells were visualized by immunostaining for spike protein and quantified by automated counting. Datapoints are means of counts from six replicate cultures ± SEM and represent % inhibition, determined as % SARS-CoV-2 positive cells relative to means of counts from infected-nontreated control cultures, and was calculated as 100%–% residual infectivity. The tested inhibitor concentration did not affect cell viability (Figure S2).

To investigate the mechanism of action of rimantadine, the most promising compound in this study, we carried out a time-of-addition experiment in VeroE6 cells. Cells were inoculated with SARS-CoV-2 during a 2-hour infection phase and treated with rimantadine at different timepoints post inoculation. When rimantadine was added at the time of inoculation (0 h post inoculation) and removed at the end of the viral infection phase (2 h post inoculation), 53% inhibition of SARS-CoV-2 infection was observed (Figure 4). However, when rimantadine was added at different timepoints following the viral infection phase (2, 4, or 6 h post viral inoculation), >99% inhibition was observed. Thus, it appeared that rimantadine acted mainly by targeting the virus at the post-entry level while partially acting at the entry level.

## 4. Discussion

We assessed the efficacy of amantadine, memantine and rimantadine, approved for the treatment of influenza A and for treatment of neurodegenerative diseases, against SARS-CoV-2 in the relevant in vitro systems, including human cell lines. While all EC50 were in the micromolar range (26–118 µM), rimantadine seemed the most favorable inhibitor with the lowest EC50 and the highest SI in all cell lines. Inhibitors showed mostly antagonistic effects in combination with remdesivir and had similar barriers to viral escape. Rimantadine acted mainly at the post-entry level.

In vitro cytotoxicity precluded the application of suppressive inhibitor concentrations in longer-term treatment assays. It is possible that the observed substitutions that the virus acquired during treatment contributed to viral escape from treatment at the given concentration by causing resistance. For example influenza A virus was shown to gain resistance during treatment with amantadine and rimantadine by acquisition of substitutions in the targets of these inhibitors [18,19,20]. Alternatively, the acquired substitutions might lead to an increase in viral fitness thus facilitating viral escape [21]. Future studies are needed to investigate the effect of the acquired substitutions.

A comparison of EC50 values with peak plasma concentrations (Cmax) and tissue concentrations might help predicting the possible clinical relevance of the investigated inhibitors. Cmax/EC50 ratios, based on EC50 values determined in this study in the different cell lines were <0.1 (Table S4). However, amantadine, showing clinical activity against influenza A, had in vitro EC50 of 16 µM [22], comparable to EC50 reported in this study. Moreover, adamantane derivatives appear to largely distribute to the lungs, with lung tissue concentrations above Cmax (Table S4). Finally, inhibitor concentrations in the respiratory tract might be further increased by the improvement of formulations allowing topical administration by inhalation [23]. Therefore, while cell culture systems provide a useful tool for determining antiviral effect and the potency of compounds in a time- and cost-effective manner, future in vivo studies would be useful to determine the clinical potential of ion-channel inhibitors for the treatment of COVID-19. To date only case reports and one observational study in patients treated with adamantane derivatives suggested potential protective effects of these drugs against COVID-19 [11,24].

Antiviral treatment is often based on combinations of drugs to increase the barrier to viral escape and to improve efficacy. Unfortunately, the combination of adamantane derivatives and remdesivir did not result in added effects or synergism. Further studies would be needed to uncover mechanisms underlying the observed antagonistic interactions. Additionally, future studies might investigate interactions of ion-channel inhibitors with other drug classes, for example protease inhibitors [14].

Future studies should investigate the mechanism of action and SARS-CoV-2 targets of the tested inhibitors. In vitro studies suggested amantadine to target SARS-CoV-2 protein E, which was suggested to have ion-channel function [6,25]. Further, amantadine was shown to inhibit the expression of a lysosomal gene involved in the viral entry of SARS-CoV-2 in vitro [26]. Moreover, molecular docking studies predicted the binding of amantadine and memantine to several SARS-CoV-2 targets including SARS-CoV-2 protein E [27,28], of amantadine to the receptor binding domain of SARS-CoV-2 spike protein [29], and of rimantadine to the 3CL protease [30]. Finally, it was suggested that amantadine and memantine had therapeutic effects on COVID-19 by modulation of the host-immune response [31]. For rimantadine, preliminary mechanism of action studies carried out in this study suggested main activity at the post-entry level and partial activity at the entry level, which might suggest that different viral targets exist.

In line with our findings, a recent report demonstrated the activity of amantadine against SARS-CoV-2 in VeroE6 cells with EC50 of 83–119 µM [32]. Our study adds novel knowledge to the field by also investigating rimantadine and memantine, both showing potency superior to amantadine. Furthermore, we demonstrated the activity of these compounds in human cell lines, including a human lung cell line. In addition, we evaluated interactions of adamantane derivatives with remdesivir, their barrier to viral escape and mode of action.

In conclusion, we report in vitro efficacy of approved clinically available ion-channel inhibitors against SARS-CoV-2. Further studies are needed to assess the clinical utility of these drugs in the fight against COVID-19.

## Figures and Tables

**Table 1:** Potency, cytotoxicity and selectivity indexes of ion-channel inhibitors in vitro.

VeroE6 cells	EC50 (µM) ^a^	CC50 (µM) ^b^	SI ^c^
**Inhibitor**			
Amantadine	116	1411	12.2
Memantine	80	611	7.6
Rimantadine	36	621	17.3
**Huh7.5 cells**	**EC50 (µM) ^a^**	**CC50 (µM) ^b^**	**SI ^c^**
**Inhibitor**			
Amantadine	118	659	5.6
Memantine	86	218	2.5
Rimantadine	26	159	6.1
**A549-hACE2 cells**	**EC50 (µM) ^a^**	**CC50 (µM) ^b^**	**SI ^c^**
**Inhibitor**			
Amantadine	80	>1429	>17.9
Memantine	70	591	8.4
Rimantadine	70	1374	19.6

^a^ EC50, median effective concentration against SARS-CoV-2. ^b^ CC50, median cytotoxic concentration; for amantadine in A549-hACE2 cells, >50% cell viability was observed at the highest concentrations tested. ^c^ SI, selectivity index, CC50/EC50.

## Data Availability

All relevant data are presented in the manuscript.

## Supplementary Materials

The following are available online at https://www.mdpi.com/article/10.3390/v13102082/s1, Figure S1: Structural formulas of ion-channel inhibitors investigated in this study. Figure S2: Cytotoxicity of ion-channel inhibitors in VeroE6 cells. Figure S3: Cytotoxicity of ion-channel inhibitors in Huh7.5 cells. Figure S4: Cytotoxicity of ion-channel inhibitors in A549-hACE2 cells. Figure S5: Analysis of interactions of ion-channel inhibitors with remdesivir in Compusyn. Figure S6: Cytotoxicity of ion-channel inhibitors in combination with remdesivir in VeroE6 cells. Table S1: Treatment conditions and residual infectivity data from combination treatment assays. Table S2: Analysis of interactions of ion-channel inhibitors with remdesivir in Compusyn. Table S3: Substitutions acquired during treatment identified in SARS-CoV-2 escape cultures. Table S4: Pharmacokinetics of adamantane derivatives.

## Abbreviations

CC50	median cytotoxic concentration(s)
CI	combination index(es)
Cmax	peak plasma concentration
COVID-19	coronavirus disease 2019
DMSO	dimethyl sulfoxide
EC50	median effective concentration(s)
HCV	hepatitis C virus
hACE2	human angiotensin-converting enzyme 2
Huh7.5	human hepatoma Huh7.5 cells
NGS	next generation sequencing
SARS-CoV-2	severe acute respiratory syndrome coronavirus 2
SEM	standard error of the mean(s)
SI	selectivity index(es)

## References

[1] Johns Hopkins University COVID-19 Dashboard by the Center for Systems Science and Engineering (CSSE) at Johns Hopkins University (JHU). https://coronavirus.jhu.edu/map.html.

[2] Zhou P., Yang X.L., Wang X.G., Hu B., Zhang L., Zhang W., Si H.R., Zhu Y., Li B., Huang C.L. (2020). A Pneumonia Outbreak Associated with a New Coronavirus of Probable Bat Origin. Nature.

[3] Beigel J.H., Tomashek K.M., Dodd L.E., Mehta A.K., Zingman B.S., Kalil A.C., Hohmann E., Chu H.Y., Luetkemeyer A., Kline S. (2020). Remdesivir for the Treatment of Covid-19—Final Report. N. Engl. J. Med..

[4] Alves Galvão M.G., Rocha Crispino Santos M.A., Alves da Cunha A.J.L. (2014). Amantadine and Rimantadine for Influenza A in Children and the Elderly. Cochrane Database Syst. Rev..

[5] Griffin S.D.C., Beales L.P., Clarke D.S., Worsfold O., Evans S.D., Jaeger J., Harris M.P.G., Rowlands D.J. (2003). The P7 Protein of Hepatitis C Virus Forms an Ion Channel That Is Blocked by the Antiviral Drug, Amantadine. FEBS Lett..

[6] Torres J., Maheswari U., Parthasarathy K., Ng L., Liu D.X., Gong X. (2007). Conductance and Amantadine Binding of a Pore Formed by a Lysine-Flanked Transmembrane Domain of SARS Coronavirus Envelope Protein. Protein Sci..

[7] Chen F., Chan K.H., Jiang Y., Kao R.Y.T., Lu H.T., Fan K.W., Cheng V.C.C., Tsui W.H.W., Hung I.F.N., Lee T.S.W. (2004). In Vitro Susceptibility of 10 Clinical Isolates of SARS Coronavirus to Selected Antiviral Compounds. J. Clin. Virol..

[8] Brison E., Jacomy H., Desforges M., Talbot P.J. (2014). Novel Treatment with Neuroprotective and Antiviral Properties against a Neuroinvasive Human Respiratory Virus. J. Virol..

[9] Mathur A., Beare A.S., Reed S.E. (1973). In Vitro Antiviral Activity and Preliminary Clinical Trials of a New Adamantane Compound. Antimicrob. Agents Chemother..

[10] Payne H.R., Storz J., Henk W.G. (1990). Initial Events in Bovine Coronavirus Infection: Analysis through Immunogold Probes and Lysosomotropic Inhibitors. Arch. Virol..

[11] Rejdak K., Grieb P. (2020). Adamantanes Might Be Protective from COVID-19 in Patients with Neurological Diseases: Multiple Sclerosis, Parkinsonism and Cognitive Impairment. Mult. Scler. Relat. Disord..

[12] Parsons C.G., Gilling K. (2007). Memantine as an Example of a Fast, Voltage-Dependent, Open Channel N-Methyl-D-Aspartate Receptor Blocker. Methods Mol. Biol..

[13] Ramirez S., Fernandez-Antunez C., Galli A., Underwood A., Pham L.V., Ryberg L.A., Feng S., Pedersen M.S., Mikkelsen L.S., Belouzard S. (2021). Overcoming Culture Restriction for SARS-CoV-2 in Human Cells Facilitates the Screening of Compounds Inhibiting Viral Replication. Antimicrob. Agents Chemother..

[14] Gammeltoft K.A., Zhou Y., Duarte Hernandez C.R., Galli A., Offersgaard A., Costa R., Pham L.V., Fahnøe U., Feng S., Scheel T.K.H. (2021). Hepatitis C Virus Protease Inhibitors Show Differential Efficacy and Interactions with Remdesivir for Treatment of SARS-CoV-2 in Vitro. Antimicrob. Agents Chemother..

[15] Zhou Y., Gilmore K., Ramirez S., Settles E., Gammeltoft K.A., Pham L.V., Fahnøe U., Feng S., Offersgaard A., Trimpert J. (2021). In Vitro Efficacy of Artemisinin-Based Treatments against SARS-CoV-2. Sci. Rep..

[16] Chou T.C., Talalay P. (1984). Quantitative Analysis of Dose-Effect Relationships: The Combined Effects of Multiple Drugs or Enzyme Inhibitors. Adv. Enzyme Regul..

[17] Chou T.-C., Martin N. (2007). CompuSyn Software for Drug Combinations and for General Doseeffect Analysis, and User’s Guide.

[18] Suzuki H., Saito R., Masuda H., Oshitani H., Sato M., Sato I. (2003). Emergence of Amantadine-Resistant Influenza A Viruses: Epidemiological Study. J. Infect. Chemother..

[19] Hayden F.G., Belshe R.B., Clover R.D., Hay A.J., Oakes M.G., Soo W. (1989). Emergence and Apparent Transmission of Rimantadine-Resistant Influenza A Virus in Families. N. Engl. J. Med..

[20] Masuda H., Suzuki H., Oshitani H., Saito R., Kawasaki S., Nishikawa M., Satoh H. (2000). Incidence of Amantadine-Resistant Influenza A Viruses in Sentinel Surveillance Sites and Nursing Homes in Niigata, Japan. Microbiol. Immunol..

[21] Serre S.B.N., Krarup H.B., Bukh J., Gottwein J.M. (2013). Identification of Alpha Interferon-Induced Envelope Mutations of Hepatitis C Virus In Vitro Associated with Increased Viral Fitness and Interferon Resistance. J. Virol..

[22] Wang J., Ma C., Balannik V., Pinto L.H., Lamb R.A., Degrado W.F. (2011). Exploring the Requirements for the Hydrophobic Scaffold and Polar Amine in Inhibitors of M2 from Influenza A Virus. ACS Med. Chem. Lett..

[23] Atmar R.L., Greenberg S.B., Quarles J.M. (1990). Safety and Pharmacokinetics of Rimantadine Small-Particle Aerosol. Antimicrob. Agents Chemother..

[24] Aranda-Abreu G.E., Aranda-Martínez J.D., Araújo R., Hernández-Aguilar M.E., Herrera-Covarrubias D., Rojas-Durán F. (2020). Observational Study of People Infected with SARS-Cov-2, Treated with Amantadine. Pharmacol. Rep..

[25] Brenner S.R., Butterworth R.F. (2020). Repurposing of Adamantanes with Transmitter Receptor Antagonist Properties for the Prevention/Treatment of COVID-19. J. Pharm. Pharmacol..

[26] Smieszek S.P., Przychodzen B.P., Polymeropoulos M.H. (2020). Amantadine Disrupts Lysosomal Gene Expression: A Hypothesis for COVID19 Treatment. Int. J. Antimicrob. Agents.

[27] Singh Tomar P.P., Arkin I.T. (2020). SARS-CoV-2 E Protein Is a Potential Ion Channel That Can Be Inhibited by Gliclazide and Memantine. Biochem. Biophys. Res. Commun..

[28] Abreu G.E.A., Aguilar M.E.H., Covarrubias D.H., Durán F.R. (2020). Amantadine as a Drug to Mitigate the Effects of COVID-19. Med. Hypotheses.

[29] Baig A.M., Khaleeq A., Syeda H. (2020). Docking Prediction of Amantadine in the Receptor Binding Domain of Spike Protein of SARS-CoV-2. ACS Pharmacol. Transl. Sci. Transl. Sci..

[30] Iftikhar H., Ali H.N., Farooq S., Naveed H., Shahzad-ul-Hussan S. (2020). Identification of Potential Inhibitors of Three Key Enzymes of SARS-CoV2 Using Computational Approach. Comput. Biol. Med..

[31] Jiménez-Jiménez F.J., Alonso-Navarro H., García-Martín E., Agúndez J.A.G.G. (2020). Anti-Inflammatory Effects of Amantadine and Memantine: Possible Therapeutics for the Treatment of Covid-19?. J. Pers. Med..

[32] Fink K., Nitsche A., Neumann M., Grossegesse M., Eisele K.H., Danysz W. (2021). Amantadine Inhibits SARS-CoV-2 in Vitro. Viruses.

