# Supplementary material for: Efficacy of Ion-Channel Inhibitors Amantadine, Memantine and Rimantadine for the Treatment of SARS-CoV-2 In Vitro
Source: Viruses. 2021 Oct 15;13(10):2082. doi: 10.3390/v13102082 (PMC8537953; doi:10.3390/v13102082)
Supplement: Supplementary file 1 [file viruses-13-02082-s001.zip › viruses-1383252-supplementary.pdf]

Supplementary Materials

Efficacy of Ion-Channel Inhibitors Amantadine, Memantine and Rimantadine for Treatment of SARS-CoV-2 in Vitro

Running title: Efficacy of Ion-Channel Inhibitors against SARS-CoV-2

Yuyong Zhou*, Karen A. Gammeltoft*, Andrea Galli, Anna Offersgaard, Ulrik Fahnøe, Santseharay Ramirez, Jens Bukh, Judith M. Gottwein[#]

Copenhagen Hepatitis C Program (CO-HEP), Department of Infectious Diseases, Copenhagen University Hospital-Hvidovre and Department of Immunology and Microbiology, Faculty of Health and Medical Sciences, University of Copenhagen, Copenhagen, Denmark

* These two authors contributed equally to this work. Author order was randomly determined.

[#] Corresponding author: jgottwein@sund.ku.dk

Supplementary Methods

Virus isolate. The corona virus isolate SARS-CoV-2/human/Denmark/DK-AHH1/2020 was isolated from a swap sample of a Danish patient by passage to VeroE6 cells. The used virus stock was derived after further passage in VeroE6 cells. The virus recovered from the previously described stock was sequence confirmed and had a SARS-CoV-2 infectivity titer of 5.5 log_{10} TCID50/mL [1].

Cells. African green monkey kidney VeroE6 cells [1] and human hepatoma Huh7.5 cells [2] were maintained in Dulbecco's Modified Eagle Medium (Invitrogen, Paisley, UK) supplemented with 10% heat inactivated fetal bovine serum (FBS) (Sigma, Saint Louis Missouri, USA), 100 U/mL penicillin and 100µg/mL streptomycin (Gibco/Invitrogen Corporation, Carlsbad, California, USA). Human lung carcinoma A549-hACE2 cells (Invivogen, Toulouse, France) were maintained in Dulbecco's Modified Eagle Medium: Nutrient Mixture F-12 (Gibco, Paisley, UK) supplemented with 10% heat inactivated FBS, 100 U/mL penicillin and 100 µg/mL streptomycin as well as 0.5 µg/mL puromycin (Invivogen, Toulouse, France) [3]. Cells were maintained at 37°C and 5% CO_2 and split every 2-3 days with trypsin (Sigma, Saint Louis, Missouri, USA) to maintain a subconfluent cell layer.

Longer-term treatments of SARS-CoV-2 infected VeroE6 cell cultures. VeroE6 cells were seeded at 10^6 cells per flask in T25 cell culture flasks (Nunc). The following day cells were infected with SARS-CoV-2/human/Denmark/DK-AHH1/2020 at MOI 0.00002, and at the same time treated with the specified fold median effective concentrations (EC50) of

inhibitors. Subsequently, cells were split and treated with the specified concentrations of inhibitors upon cell splitting, every 2-3 days. Upon cell splitting, cell culture supernatants were obtained and stored at -80^{0}C. The percentage of infected culture cells was evaluated by immunostaining for the SARS-CoV-2 spike protein and immunofluorescence imaging, as described below. Virus- or drug-induced cytopathogenic effects were graded by inspection in the light microscope assigning four different grades (no, slight, pronounced and massive). When massive virus induced cell death occurred, cultures were closed following recovery of culture supernatant. Thus, for this final culture day supernatant but not immunostaining was available.

Evaluation of longer-term treatments by immunostaining. Procedures were previously described [3]. Upon cell splitting of longer-term treatment cultures and following addition of inhibitor, replicate cultures were seeded in chamber slides (Thermo Fisher Scientific, Rochester, NY, USA). The next day cells were fixed by submersion into methanol for 20min. Cells were then incubated with primary antibody SARS-CoV-2 spike chimeric monoclonal antibody (Sino Biological #40150-D004, Beijing, China) diluted 1:1,000 followed by incubation with secondary antibody Alexa-Fluor 488 goat anti-human IgG (H+L) (Invitrogen #A-11013, Paisley, UK) diluted 1:500 and Hoechst 33342 (Invitrogen, Paisley, UK) diluted 1:1,000. The % of infected cells was evaluated by fluorescence microscopy using a Zeiss Axiovert equipped with a 40× LD Plan-Neofluar objective (NA 0.6) (Jena, Germany), relating the estimated number of SARS-CoV-2 spike protein positive

stained cells to the estimated number of Hoechst stained cells. Scores of 0% infected cells, single infected cells, 1%, 5% and 10%-90% infected cells in steps of 10% were assigned.

Next generation sequencing of SARS-CoV-2 genomes from supernatant in long-term treatment cultures. For extraction of SARS-CoV-2 RNA from cell culture supernatants as well as reverse transcription reaction (RT) and polymerase chain reaction (PCR) spanning the complete open reading frame by 5 overlapping amplicons, methodology was adapted from previously established methods for analysis of hepatitis C virus [1,4,5]. In brief, SARS-CoV-2 RNA was extracted from 100 µL cell culture supernatant with 300 µL Trizol LS (Life Technologies, Carlsbad, California, USA) and 100 µL chloroform (Sigma, Saint Louis, Missouri, USA) by use of 5PRIME Phase Lock Gel Heavy tubes (Quantabio, Beverly, Massachusetts, USA). Then RNA was purified using RNA Clean and Concentrator™ – 5 (ZYMO, Irvine, California, USA), following the manufacturer's guidelines. Lastly, RNA was eluted in 30 µL nuclease-free H_2O (Ambion, Austin, Texas, USA). RT-PCR and PCR reagents and primers as well as thermocycler conditions were as described [1]. PCR amplicons were purified using DNA Clean and Concentrator™ – 25 (ZYMO, Irvine, California, USA) following the manufacturer's guidelines and eluted in 30 µL nuclease-free H_2O. Library preparations were done using NEBNext Ultra II DNA library Prep kit (New England BioLabs, Ipswich, Massachusetts, USA) and NGS analysis was done as described [1].

Short-term concentration-response treatments for determination of ion-channel inhibitor potency. Antiviral short-term concentration-response treatment assays were

carried out as previously described [1,3,6]. VeroE6, Huh7.5 and A549-hACE2 cells were seeded in 96-well flat-bottom plates (Thermo Fischer Scientific, Roskilde, Denmark) at 10,000, 9,000 and 10,000 cells per well, respectively. The next day cells were inoculated with SARS-CoV-2/human/Denmark/DK-AHH1/2020 at MOI 0.002 for VeroE6 cells, MOI 0.02 for Huh7.5 cells, and MOI 0.003 for A549-hACE2 cells, based on the infectivity titer determined in VeroE6 cells. Following 1-hour incubation at 37°C and 5% CO_2 infected cells were treated with a dilution series of ion-channel inhibitors, or a dilution series of DMSO alone serving as a control for antiviral activity of DMSO. All treatment concentrations were tested in 7 replicates. Additionally, as all inhibitors were diluted in DMSO, inhibitor concentrations at which corresponding DMSO dilutions were expected to cause antiviral effects (residual infectivity < 70%) were excluded based on previously obtained results [3]. All treatment plates included 14 infected-nontreated wells serving as a positive control for infection, and 12 noninfected-nontreated blank wells serving as a negative control for infection. For A549-hACE2 cells, the concentration of DMSO was kept constant in all cultures. After 46-50 hours for VeroE6 and A549-hACE2 cells, and after 70-74 hours for Huh7.5 cells, plates were subjected to immunostaining for the SARS-CoV-2 spike protein and evaluated as described below.

Short-term concentration-response combination treatments for analysis of interactions of ion-channel inhibitors with remdesivir. Interactions of ion-channel inhibitors and remdesivir for inhibition of SARS-CoV-2 were investigated as previously described [3]. The experimental setup was similar to that of short-term treatments with single ion-

channel inhibitors described above. In brief, VeroE6 cells were seeded at 10,000 cells per well in 96-well flat-bottom plates. The next day cells were inoculated with SARS-CoV-2/human/Denmark/DK-AHH1/2020 at MOI 0.002. Following 1-hour incubation at 37°C and 5% CO_2, infected cells were treated with a dilution series of inhibitors. Dilution series of ion-channel inhibitors singly, remdesivir singly or a combination of ion-channel inhibitors and remdesivir were chosen based on previously determined EC50 values against SARS-CoV-2 in VeroE6 cells (see Figure 1 and Table 1 for ion-channel inhibitors as well as Gammeltoft and Zhou et al. for remdesivir [3]). Thus, for inhibitors and combinations of inhibitors infected cells were treated with a 1.5-fold dilution series with 10 different dilutions spanning the respective EC50 values and aiming at achieving residual infectivities between 0 and 100 %. The same ion-channel inhibitor concentrations and remdesivir concentrations as used in single treatments were applied in the combination treatments; inhibitors were combined at a fixed ratio. All treatment conditions were tested in 7 replicates including 14 infected-nontreated replicates and 12 noninfected-nontreated replicates per experimental plate. After 46-50 hours incubation, plates were subjected to immunostaining for the SARS-CoV-2 spike protein and evaluated as described below.

Time-of-addition experiment with rimantadine. The time-of-addition experiment was carried out as previously described [6]. VeroE6 cells were seeded in 96-well flat-bottom plates (Thermo Fischer Scientific, Roskilde, Denmark) at 10,000 cells per well. The next day cells were inoculated with SARS-CoV-2/human/Denmark/DK-AHH1/2020 at MOI 0.01 for a 2-hour infection phase. Following this infection phase, virus containing medium was

removed and wells were washed one time with PBS, followed by addition of fresh growth medium. 230 μM rimantadine was added at different timepoints post inoculation. For viral entry treatment, inhibitor was added together with the virus, at 0-hour post inoculation and removed together with the virus following the 2-hour infection phase. For post-entry treatment, inhibitor was added 2, 4 or 6 hours post inoculation and maintained throughout the experiment. Each treatment condition was tested in 6 replicates, and the experimental plate included 12 infected-nontreated cultures and 12 noninfected-nontreated cultures. After 46-50 hours the plate was subjected to immunostaining for the SARS-CoV-2 spike protein and evaluated as described below.

Immunostaining and evaluation of 96-well plates for short-term treatment assays with single inhibitors, and inhibitor combinations as well as for time-of-addition experiment. These procedures were carried out as previously described [1,3,6]. Cells were fixated and virus was inactivated by submersion into methanol (J.T.Baker, Gliwice, Poland) and incubation for 20 minutes at room temperature. For immunostaining of the SARS-CoV-2 spike protein, plates were first washed with PBS-tween [PBS (Sigma, Gillingham, UK) containing 0.1% Tween-20 (Sigma, Saint Louis, Missouri)] and blocked for endogenous peroxidase activity by addition of H_2O_2 and incubation for 10 minutes. This was followed by additional washing with PBS-tween and blocking by PBSK [PBS containing 1% bovine serum albumin (Roche, Mannheim, Germany) and 0.2% skim milk (Easis, Aarhus, Denmark)] for 30 minutes. Plates were then incubated with primary antibody SARS-CoV-2 spike chimeric monoclonal antibody (Sino Biological #40150-D004,

Beijing, China) diluted 1:5,000 in PBSK for 2 hours at room temperature. Then plates were washed with PBS-tween and incubated with secondary antibody F(ab')2-Goat anti-human IgG Fc Cross-Adsorbed Secondary Antibody, HRP (Invitrogen#A24476, Carlsbad, CA, USA) or Goat F(ab')2 Anti-Human IgG – Fc (HRP), preadsorbed (Abcamab#98595, Cambridge, UK), diluted 1:2,000 in PBSK. Lastly, plates were washed with PBS-tween, and SARS-CoV-2 spike protein positive cells were stained using DAB substrate BrightDAB kit (Immunologic # BS04-110, Duiven, Netherlands) according to the manufacturer's guidelines. Plates were evaluated by automated counting of single SARS-CoV-2 spike protein positive cells using an ImmunoSpot series 5 UV Analyzer (CTL Europe GmbH, Bonn, Germany). Mean of counts of infected-nontreated wells were 3,000-4,000 for VeroE6 cells, 1,000-2,000 for Huh7.5 cells and 2,000-3,000 for A549-hACE2 cells. The mean of counts from noninfected-nontreated wells was subtracted from counts of infected wells; this mean was typically lower than 50 counts. Counts from infected and treated wells were related to the mean count of the 14-replicate infected-nontreated wells to calculate % residual infectivity as:

100×(count in infected-treated culture/mean of counts in infected-nontreated cultures). Datapoints are given as means of 7 replicates with standard error of the means (SEM). Sigmoidal concentration-response curves were fitted and EC50 values were calculated using Graphpad Prism 8.0.0 with a bottom constraint of 0 applying the formula $Y= Top/(1+10^{(Log10EC50-X)*HillSlope})$ [1,3,6]. Representative images from concentration-response antiviral treatment assays are shown in Zhou and Gilmore et al [6].

Interaction of ion-channel inhibitors with remdesivir was further investigated using the method of Chou and Talalay [7] and CompuSyn freeware (ComboSyn Inc) [8]. % inhibition values were calculated as (1-residual infectivity), which were designated fractional effect (Fa) values, ranging from 0.01 to 0.99, and which were entered into CompuSyn software. For each inhibitor or inhibitor combination 10 datapoints were entered based on 10 tested concentrations. The software was then used to determine (i) concentration-effect curves for single and combination treatments, (ii) combination index (CI) values and curves in relation to Fa values, and (iii) drug reduction index (DRI) values and curves in relation to Fa values. CI values were used to grade level of synergy and antagonism as suggested by CompuSyn: CI<0.1, very strong synergism; 0.1≤CI<0.3, strong synergism; 0.3≤CI<0.7, synergism; 0.7≤CI<0.85, moderate synergism; 0.85≤CI<0.9, slight synergism; 0.9≤CI<1.1, nearly additive; 1.1≤CI<1.2, slight antagonism; 1.2≤CI<1.45, moderate antagonism; 1.45≤CI<3.3, antagonism; 3.3≤CI<10, strong antagonism; CI≥10, very strong antagonism. DRI values describe how many folds the concentration of each inhibitor could be reduced due to synergism during combination treatment and at given levels of inhibition, compared to each inhibitor administered alone.

For evaluation of the time-of-addition experiment, counts from infected and treated wells were related to the mean count of the 12-replicate infected-nontreated wells to calculate % inhibition. % inhibition was calculated as 100% - % residual infectivity. Datapoints are given as means of 6 replicates with SEM. An image from the time-of-addition experiment is shown in Figure S7.

Cell viability concentration response assays. To evaluate cytotoxic effects of the inhibitors cell viability was monitored using a colorimetric assay, as previously described [3,6]. VeroE6, Huh7.5 and A549-hACE2 cells were seeded at 10,000, 9,000 and 10,000 cells per well, respectively, in 96-well flat-bottom plates. The following day cells were treated with dilution series of inhibitors. After 46-50 hours for VeroE6 and A549-hACE2 cells and after 70-74 hours for Huh7.5 cells, cell viability was evaluated using the CellTiter 96 Aqueous One Solution Cell Proliferation Assay (Promega, Madison, WI, USA) following the manufacturer's guidelines. In brief, 20 µL CellTiter 96 Aqueous One Solution Reagent was added to each well and plates were then incubated for 1 to 3 hours at 37°C and 5% CO_2. After incubation, for each well absorbance at 492 nm was recorded by use of a FLUOstar OPTIMA 96-well plate reader (BMG, LABTECH, Offenburg, Germany). Each inhibitor concentration was tested in 3 to 4 replicate wells and each experimental plate included 12 replicate nontreated control wells. Absorbance values of treated wells were related to the mean absorbance of the nontreated wells to estimate % cell viability. Datapoints are given as means of 3 to 4 replicates with SEM. Sigmoidal concentration-response curves were fitted and median cytotoxic concentration (CC50) values were calculated using GraphPad Prism 8.0.0 with a bottom constraint of 0 applying the formula $Y= Top/(1+10^{(Log10EC50-X)*HillSlope})$.

Supplementary Results

Amantadine, memantine and rimantadine interacted in a similar antagonistic manner with remdesivir. To determine interactions of adamantane derivatives with remdesivir, the only approved drug for the treatment of COVID-19 directly targeting a SARS-CoV-2 protein, we carried out short-term combination treatment assays on 96-well plates. Based on evaluation of concentration-response curves, for all tested ion-channel inhibitors the effect of the combination treatment did not exceed the effect of the single treatments, thus for example for amantadine at 73.2 μM and remdesivir at 1.7 μM, single treatments resulted in 80% and 60%, residual infectivity, respectively, while the combination treatment resulted in 59% residual infectivity (Figure 2 and Table S1).

Further analysis of the generated data using the software CompuSyn [8], based on a method developed by Chou and Thalalay [7], revealed mostly antagonistic interactions (Figure S5 and Table S2). Fa-CI plots suggested antagonism of ion-channel inhibitors with remdesivir at all key Fa values, with CI values being at least 1.6 (Figure S5 B, C and Table S2). In more detail, for amantadine + remdesivir, at key Fa values antagonism was suggested by CI values of 1.8. At Fa values of 0.02 to 0.99 at experimental datapoints mostly antagonism was suggested by CI values of 1.5 to 2.6. For memantine + remdesivir at key Fa values antagonism was suggested by CI values of 1.6 to 1.8 and at Fa values of 0.03 to 0.99 at experimental datapoints mostly antagonism was suggested by CI values of 1.5 to 2.7. For rimantadine + remdesivir at key Fa values antagonism was suggested by CI values of 1.6 to 1.9 and at Fa values of 0.01 to 0.99 at experimental datapoints mostly

antagonism was suggested by CI values of 1.7 to 2.7. In agreement with the observed antagonism, combinations of ion-channel inhibitors with remdesivir suggested little to no drug reduction potential with most DRI values close to 1 (Figure S5 D and Table S2). The combinations of inhibitors used in this assay did not cause cytotoxic effects (cell viability>90%) (Figure S6).

Supplementary Figures

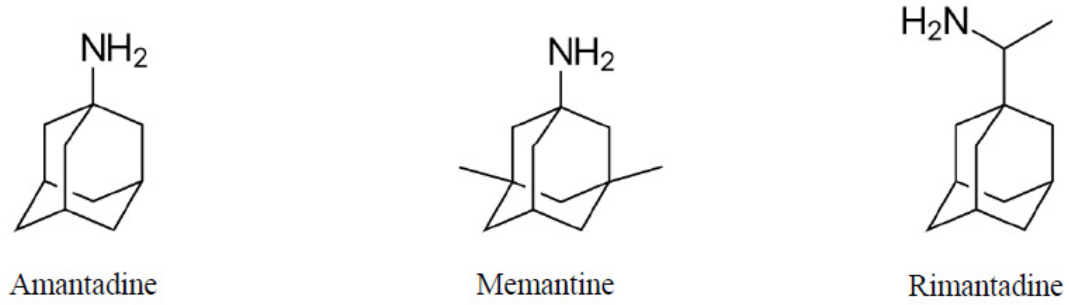

Figure S1. Structural formulas of ion-channel inhibitors investigated in this study

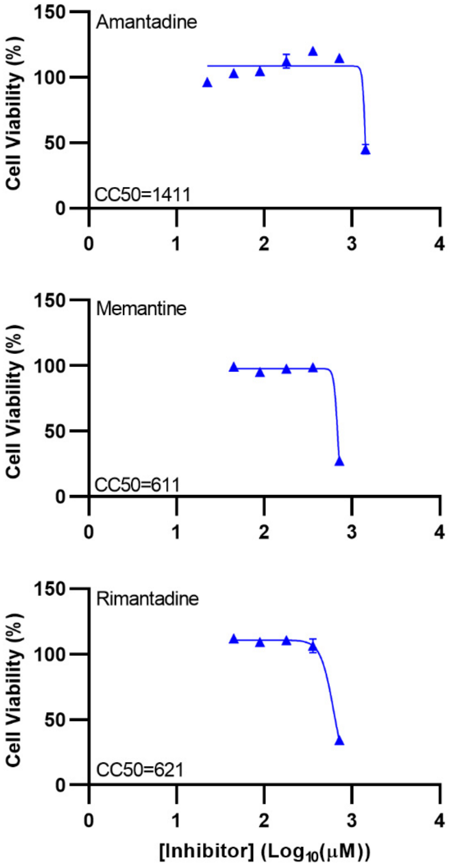

Figure S2. Cytotoxicity of ion-channel inhibitors in VeroE6 cells. Cell viability data were obtained in treatment assays in noninfected VeroE6 cells using a colorimetric assay. Data points represent % cell viability relative to the mean of 12 nontreated controls and are given as means of 3-4 replicates with SEM. Sigmoidal concentration response curves were fitted and CC50 values were determined using Graphpad Prism 8.0.0 applying the formula Y= Top/(1+10$^{(Log10EC50-X)*HillSlope}$) with a bottom constrain of 0. For all assays, additional data points on the lower plateau were excluded to improve curve fitting and

determination of CC50 values; thus, only the lowest inhibitor concentration defining the lower plateau was included.

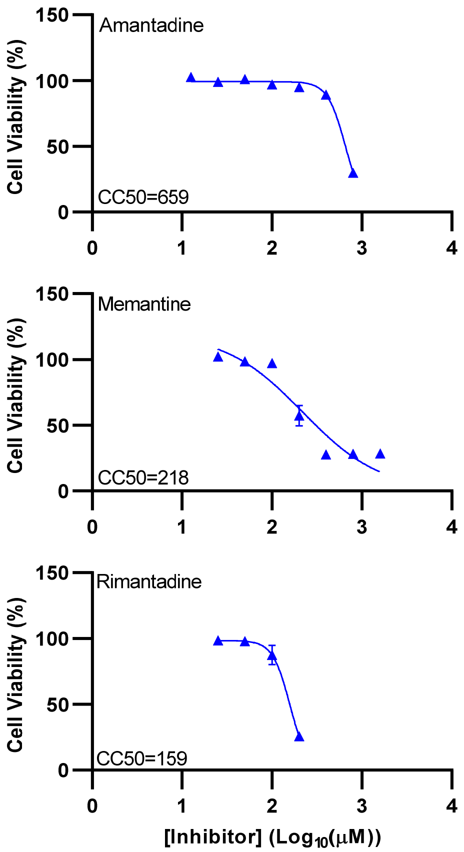

Figure S3. Cytotoxicity of ion-channel inhibitors in Huh7.5 cells. Cell viability data were obtained in treatment assays with noninfected Huh7.5 cells using a colorimetric assay. Data points represent % cell viability relative to the mean of 12 nontreated controls and are given as means of 3-4 replicates with SEM. Sigmoidal concentration response curves were fitted and CC50 values were determined using Graphpad Prism 8.0.0 applying the formula $Y= Top/(1+10^{(Log10EC50-X)*HillSlope})$ with a bottom constrain of 0. For all assays,

additional data points on the lower plateau were excluded to improve curve fitting and determination of CC50 values; thus, only the lowest inhibitor concentration defining the lower plateau was included.

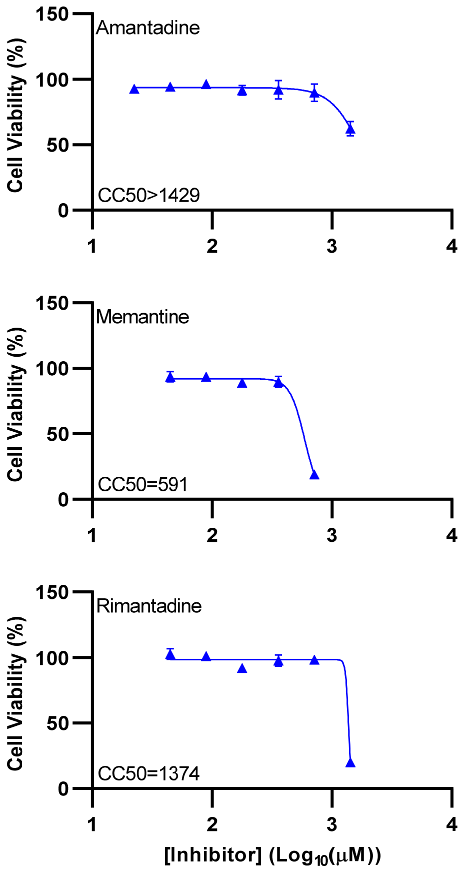

Figure S4. Cytotoxicity of ion-channel inhibitors in A549-hACE2 cells. Cell viability data were obtained in treatment assays with noninfected A549-hACE2 cells using a colorimetric assay. Data points represent % cell viability relative to the mean of 12 nontreated controls and are given as means of 3-4 replicates with SEM. Sigmoidal concentration response curves were fitted and CC50 values were determined using Graphpad Prism 8.0.0

applying the formula $Y= Top/(1+10^{(Log10EC50-X)*HillSlope})$ with a bottom constrain of 0. For all assays, additional data points on the lower plateau were excluded to improve curve fitting and determination of CC50 values; thus, only the lowest inhibitor concentration defining the lower plateau was included.

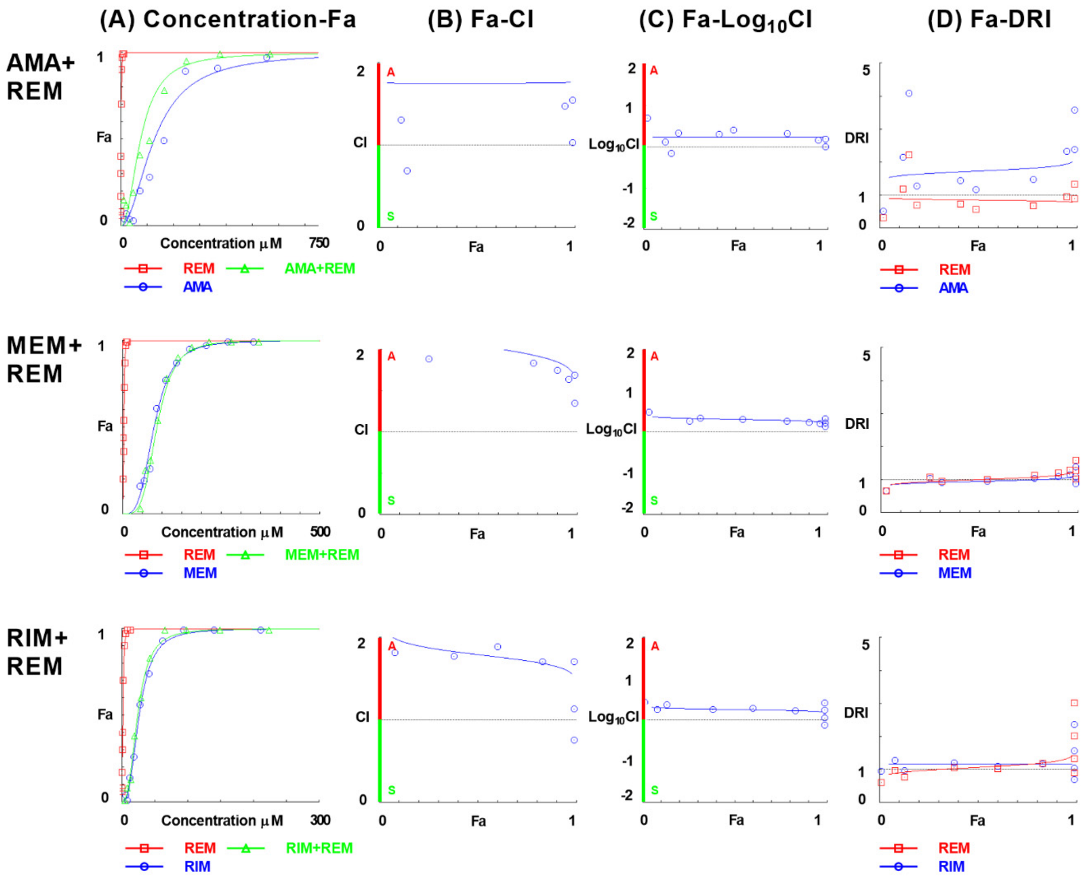

Figure S5. Analysis of interactions of ion-channel inhibitors with remdesivir.

VeroE6 cells seeded in 96 well plates were infected with SARS-CoV-2 and treated with specified concentrations of ion-channel inhibitors amantadine (AMA), memantine (MEM), rimantadine (RIM) or polymerase inhibitor remdesivir (REM) or a combination of ion-

channel inhibitor and remdesivir. SARS-CoV-2 infected cells were visualized by immunostaining for SARS-CoV-2 spike protein and quantified by automated counting. Datapoints in panel A represent Fractional effect (Fa) values which were calculated by relating counts from infected and treated cultures to the mean count from infected-nontreated cultures, which were entered into CompuSyn software. Datapoints from representative experiments are given as means of 7 replicates and for each treatment 10 datapoints were entered. Combination index (CI) and drug reduction index (DRI) values were calculated in CompuSyn based on the above described Fa values. For each inhibitor combination, the following curves were fitted using Compusyn: (A) concentration-Fa curves plotting Fa values ranging from 0.01 to 0.99 against specified inhibitor concentrations. (B) Fa-CI curves plotting CI values ranging from 0 to 2 against Fa values ranging from 0.01 to 0.99. (C) Fa-Log_{10}CI curves plotting logarithmic CI values ranging from 0.01 to 100 against Fa values ranging from 0.01 to 0.99. (B and C) Overall, CI values ≥1.1 suggest antagonism "A", while CI values <0.9 suggest synergism "S" indicated by green and red colour coding of the y-axis, respectively. (D) Fa-DRI curves plotting DRI values ranging from 0 to 5 against Fa values ranging from 0.01 to 0.99.

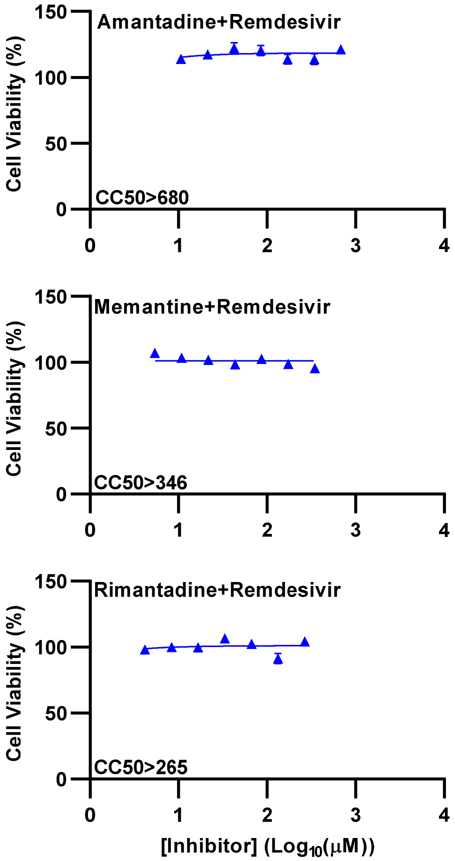

Figure S6. Cytotoxicity of ion-channel inhibitors in combination with remdesivir in VeroE6 cells. Cell viability data were obtained in treatment assays with noninfected VeroE6 cells using a colorimetric assay. Data points represent % cell viability relative to the mean of 12 nontreated controls and are given as means of 3-4 replicates with SEM. Concentrations are given as total concentration of inhibitor ([ion-channel inhibitor]+[remdesivir]). Sigmoidal concentration response curves were fitted and CC50 values were determined using Graphpad Prism 8.0.0 applying the formula Y= Top/(1+10$^{(Log10EC50-X)*HillSlope}$) with a bottom constrain of 0.

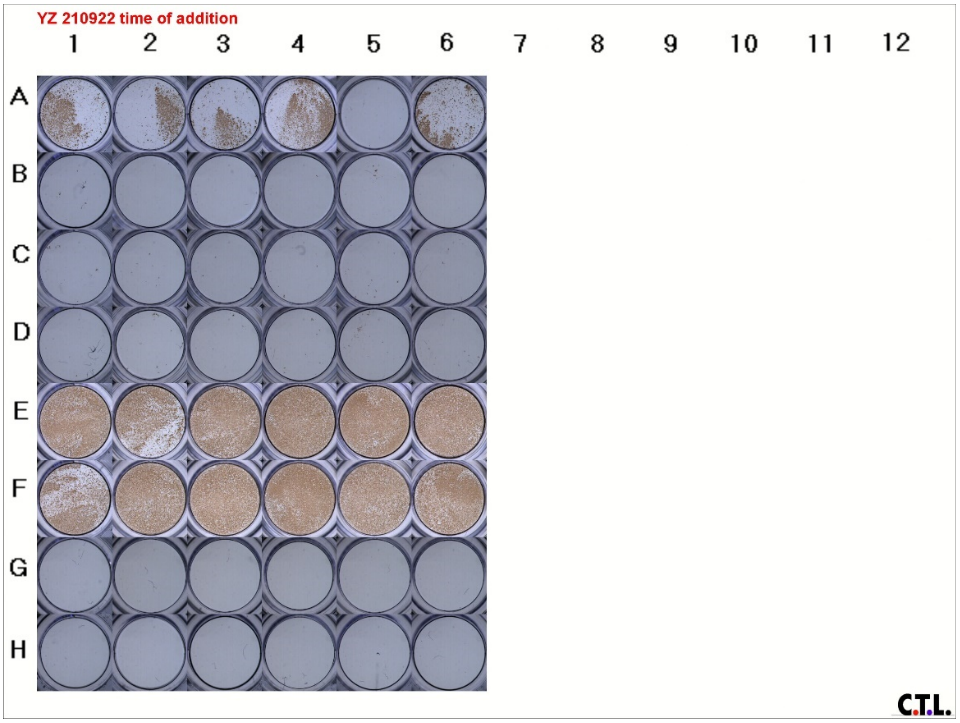

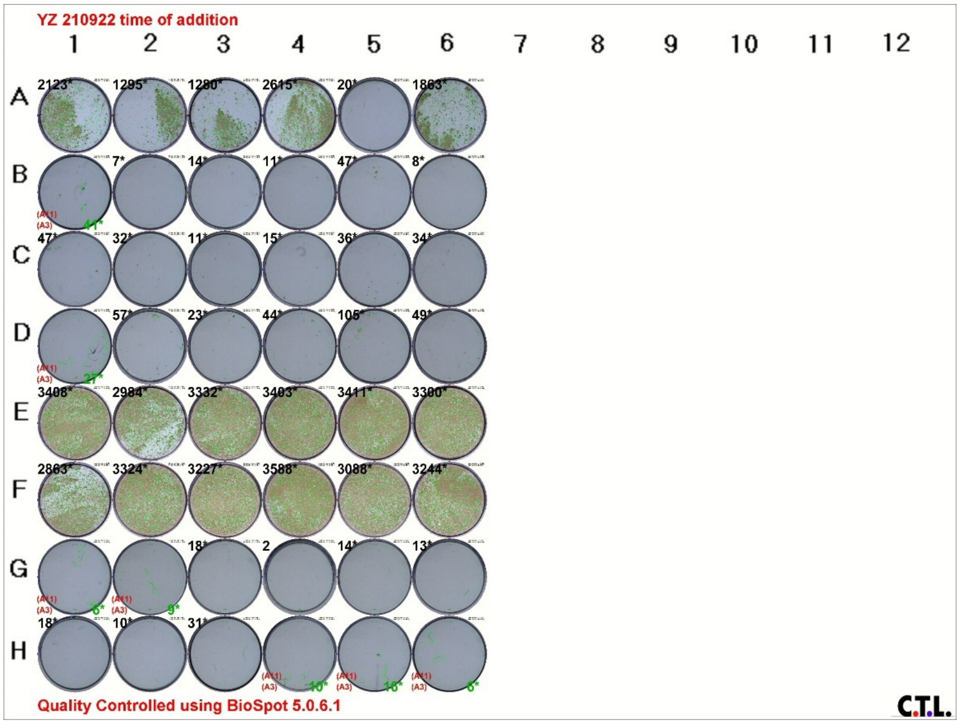

Figure S7. Image of 96-well plate from time-of-addition experiment in VeroE6 cells. The time-of-addition experiment was carried out in VeroE6 cells by inoculating with SARS-CoV-2 for a 2-hour infection phase and treating with rimantadine at different timepoints

post viral inoculation. The upper panel shows the experimental plate post staining, before automated counting. The lower panel shows the experimental plate post staining and post automated counting of SARS-CoV-2 infected cells. Each treatment condition was tested in 6 replicates and the experiment included 12 infected-nontreated and 12 noninfected-nontreated control cultures. The identity of cultures on the shown plate image is as follows:A1-A6: Rimantadine addition together with the virus (0 hour post inoculation) and rimantadine presence during the 2-hour viral infection phase; B1-B6: Rimantadine addition 2 hours post viral inoculation and presence throughout the experiment; C1-C6: Rimantadine addition 4 hours post viral inoculation and presence throughout the experiment; D1-D6: Rimantadine addition 6 hours post viral inoculation and presence throughout the experiment; E1-E6, F1-F6: Infected-nontreated control cultures; G1-G6, H1-H6: Noninfected-nontreated control cultures.

Supplementary Tables

Table S1. Treatment conditions and residual infectivity data from combination treatment assay.

Treatment[a]	Concentration (μM)[b]		Residual Infectivity (%)[c]		
	AMA	REM	AMA	REM	Combi
1	14.5	0.3	96	94	85
2	21.7	0.5	93	92	88
3	32.5	0.8	96	83	98
4	48.8	1.1	97	70	81
5	73.2	1.7	80	60	59
6	109.7	2.5	72	30	51
7	164.6	3.8	51	10	22
8	246.9	5.7	31	3	5
9	370.4	8.6	9	0	0
10	555.6	12.9	3	1	-1
Ratio[d]	43	1			
**Treatment[a]**	**Concentration (μM)[b]**		**Residual Infectivity (%)[c]**		
	MEM	REM	MEM	REM	Combi
1	44.7	1.1	84	80	97
2	55.9	1.4	81	80	75
3	69.9	1.8	74	56	69
4	87.4	2.2	39	64	46
5	109.2	2.8	23	46	22
6	136.5	3.5	13	27	10
7	170.7	4.3	5	13	4
8	213.3	6.5	3	3	1
9	266.7	9.7	1	1	0
10	333.3	12.2	1	1	1
Ratio[d]	27	1			
**Treatment[a]**	**Concentration (μM)[b]**		**Residual Infectivity (%)[c]**		
	RIM	REM	RIM	REM	Combi
1	5.5	0.3	96	94	101
2	8.2	0.5	101	92	92
3	12.4	0.8	86	83	87
4	18.5	1.1	74	70	62
5	27.8	1.7	44	60	40
6	41.7	2.5	26	30	17
7	62.6	3.8	7	10	0
8	93.8	5.7	1	3	-1
9	140.7	8.6	1	0	-2
10	211.1	12.9	1	1	0
Ratio[d]	16	1			

[a]Treatment, number of treatment condition and the specified concentrations of inhibitors. Infected cultures were treated with inhibitors either singly or in combination in antiviral treatment assays for analysis of interactions; results of these assays are shown in Figure 2.

[b] Concentration (μM), concentrations of inhibitors, AMA, amantadine, MEM, memantine, RIM, rimantadine, REM, remdesivir.

[c] Residual infectivity (%), calculated by relating the number of infected cells in treated cultures to the mean number of infected cells in infected-nontreated control cultures. Residual infectivity is given for single treatments with the specified inhibitors and the respective combination treatments (Combi) for each treatment condition.

[d] Ratio, for all inhibitor combinations a fixed concentration ratio was applied as specified.

Table S2. Analysis of interactions of ion-channel inhibitors with remdesivir in Compusyn.

	CI[a]				DRI[b]					
	Key Fa[c]		Experimental Fa[b]		Key Fa[c]			Experimental Fa[d]		
						AMA	REM		AMA	REM
	Key Fa	CI	Exp. Fa	CI	Key Fa	DRI	DRI	Exp. Fa	DRI	DRI
	0.25	1.8	0.02	5.1	0.25	1.7	0.9	0.02	0.5	0.3
	0.5	1.8	0.12	1.3	0.5	1.7	0.9	0.12	2.1	1.2
**AMA+REM**	0.75	1.8	0.15	0.7	0.75	1.8	0.8	0.15	4.1	2.2
	0.9	1.8	0.19	2.2	0.9	1.9	0.8	0.19	1.3	0.7
			0.41	2.1				0.41	1.4	0.7
			0.49	2.6				0.49	1.2	0.6
			0.78	2.2				0.78	1.5	0.7
			0.95	1.5				0.95	2.3	1.0
			0.99	1.0				0.99	3.6	1.3
			0.99	1.5				0.99	2.4	0.9
						MEM	REM		MEM	REM
	0.25	1.8	0.03	2.7	0.25	1.4	1.0	0.03	1.0	0.6
	0.5	1.7	0.25	1.6	0.5	1.4	1.0	0.25	1.5	1.1
	0.75	1.7	0.31	1.9	0.75	1.3	1.1	0.31	1.3	0.9
	0.9	1.6	0.54	1.7	0.9	1.3	1.2	0.54	1.4	1.1
**MEM+REM**			0.78	1.5				0.78	1.5	1.2
			0.9	1.4				0.9	1.6	1.4
			0.96	1.2				0.96	1.7	1.6
			0.99	1.2				0.99	1.7	1.7
			0.99	1.8				0.99	1.1	1.2
			0.99	2.2				0.99	0.9	0.9
						RIM	REM		RIM	REM
	0.25	1.9	0.01	2.7	0.25	1.2	1.0	0.01	0.9	0.6
	0.5	1.8	0.08	1.8	0.5	1.2	1.1	0.08	1.3	1.0
	0.75	1.7	0.13	2.3	0.75	1.2	1.2	0.13	1.0	0.8
	0.9	1.6	0.38	1.7	0.9	1.2	1.3	0.38	1.2	1.1
**RIM+REM**			0.6	1.9				0.6	1.1	1.0
			0.83	1.7				0.83	1.2	1.2
			0.99	0.8				0.99	2.4	3
			0.99	1.1				0.99	1.6	2
			0.99	1.7				0.99	1.0	1.3
			0.99	2.6				0.99	0.7	0.9

CI
- 0.1 ≤ CI < 0.3, strong synergism
- 0.3 ≤ CI < 0.7, synergism
- 0.7 ≤ CI < 0.85, moderate synergism
- 0.9 ≤ CI < 1.1, nearly additive
- 1.1 ≤ CI < 1.2, slight antagonism
- 1.2 ≤ CI < 1.45, moderate antagonism
- 1.45 ≤ CI < 3.3, antagonism
- 3.3 ≤ CI, strong antagonism

DRI
- DRI ≥ 20
- 10 ≤ DRI < 20
- 5 ≤ DRI < 10
- 3 ≤ DRI < 5
- 1.5 ≤ DRI < 3
- DRI < 1.5

[a] CI, Combination index values generated by analysis in CompuSyn of data from short-term treatment assays carried out for analysis of interactions of ion-channel inhibitors amantadine (AMA), memantine (MEM), rimantadine (RIM) and remdesivir (REM) (Figure 2 and Table S1). Graphical illustrations of these results are shown in Figure S5 B and C. CI are color-coded according to the legend below the table and reflect the mode of interaction. In the range 0.85≤CI<0.9 (slight synergism), no values were recorded.

[b] DRI, Drug reduction index values generated by CompuSyn analysis. DRI values are color-coded according to the legend below the table. Graphical illustrations of these results are shown in Figure S5 D.

[c] Key Fa, key fractional effects identical with key inhibition percentages (0.25, 0.5, 0.75 and 0.9).

[d] Experimental Fa, fractional effects identical with inhibition percentages achieved at experimental datapoints.

Table S3. Next generation sequencing of SARS-CoV-2 genomes from long-term treatment experiments.

Nucleotide position[a]	Reference nucleotide[b]	Nucleotide change[c]	Frequency in non-treated day 5 (%)[d]	Frequency in Amantadine 3xEC50 day 5 (%)[e]	Frequency in Memantine 3xEC50 day 5 (%)[e]	Frequency in Rimantadine 3xEC50 day 5 (%)[e]	Frequency in Rimantadine 7xEC50 day 7 (%)[e]	Amino acid change[f]	SARS-CoV-2 protein[g]
2343	T	G	-	-	-	-	99.7	I513S	nsp2
3162	C	A	-	-	21.6	-	-	S148Y	nsp3
3482	G	A	-	-	52.0	-	-	G255R	nsp3
11522	T	G	-	-	-	98.6	-	F184V	nsp6
16534	A	G	5.5	-	-	-	-	S100G	nsp13
17368	A	G	5.2	-	-	-	-	M378V	nsp13
17964	G	T	6.3	-	-	-	-	M576I	nsp13
22487	A	G	52.6	89.8	23.1	99.5	-	K309E	S

[a]Nucleotide position relating to the SARS-CoV-2/human/Denmark/DK-AHH1/2020 genome sequence (GenBank accession number MZ049597). Coding nucleotide changes occurring in at least one of the analyzed virus populations with ≥5% prevalence are listed.

[b]Nucleotide identity in the SARS-CoV-2/human/Denmark/DK-AHH1/2020 sequence.

[c]Changed nucleotide detected in cell culture derived virus.

[d]Frequency of the indicated nucleotide change in the genomes of the viral population in the nontreated culture on day 5 post infection. -, frequency of the indicated nucleotide change was below the limit of detection.

[e]Frequency of the indicated nucleotide change in the genomes in the viral populations in cultures subjected to the specified treatments at the given timepoint post infection and treatment initiation. -, frequency of the indicated nucleotide change was below the limit of detection

[f]Amino acid change encoded by the given nucleotide change. Position numbers relate to the respective protein of SARS-CoV-2/human/Denmark/DK-AHH1/2020.

[g]SARS-CoV-2 protein, where the given amino acid change is located.

Table S4. Pharmacokinetics of adamantane derivatives

Inhibitor	Cmax (μM)[a]	Cmax/EC50[b]	Dose[c]	PPB[d]	Distribution to lungs[e]	References[f]
Amantadine	2.7	0.02/0.02/0.03	200mg single dose	67%	High	*https://www.accessdata.fda.gov/drugsatfda_docs/label/2009/016023s041,018101s016lbl.pdf, Bleidner, W. E., Harmon, J. B., Hewes, W. E., Lynes, T. E. & Hermann, E. C. ABSORPTION, DISTRIBUTION AND EXCRETION OF AMANTADINE HYDROCHLORIDE. J. Pharmacol. Exp. Ther. 150, (1965)*
Memantine	0.1	0.001/0.001/0.001	20mg single dose	45%	High	*doi: 10.1007/s12325-019-01044-y, Kuns, B., Rosnai, A. & Varghese, D. Memantine - StatPearls - NCBI Bookshelf. Memantine (2020)*
Rimantadine	2.2	0.06/0.08/0.03	100mg/12hrs	40%	na	*https://www.accessdata.fda.gov/drugsatfda_docs/label/2010/019649s015lbl.pdf*

[a]Cmax, maximum plasma concentrations (μM) of ion-channel inhibitors achieved in patients after administration of specified dose(s). For rimantadine Cmax values were determined at steady state after multiple dosings.

[b]Cmax/EC50, peak plasma concentration (μM) relative to the EC50 (μM) of inhibitor against SARS-CoV-2 determined in VeroE6, Huh7.5 and A549-hACE2 cells, respectively (Figure 1, Table 1).

[c]Dose, dosing scheme leading to the reported Cmax values. All doses are in accordance to recommended clinical dosings.

[d]PPB, plasma protein binding, % of compound bound to plasma proteins according to FDA reports.

[e]Distribution of inhibitor to pulmonary tissue relative to Cmax. na, data not available.

[f]References for listed Cmax values and distribution. Values were extracted from either FDA reports or if not available, values were extracted from publications on representative clinical studies.

References

1. Ramirez, S.; Fernandez-Antunez, C.; Galli, A.; Underwood, A.; Pham, L. V.; Ryberg, L.A.; Feng, S.; Pedersen, M.S.; Mikkelsen, L.S.; Belouzard, S.; et al. Overcoming Culture Restriction for SARS-CoV-2 in Human Cells Facilitates the Screening of Compounds Inhibiting Viral Replication. *Antimicrob. Agents Chemother.* **2021**, *65*, e0009721.

2. Blight, K.J.; Mckeating, J.A.; Rice, C.M. Highly Permissive Cell Lines for Subgenomic and Genomic Hepatitis C Virus RNA Replication. *J. Virol.* **2002**, *76*, 13001–13014.

3. Gammeltoft, K.A.; Zhou, Y.; Duarte Hernandez, C.R.; Galli, A.; Offersgaard, A.; Costa, R.; Pham, L. V; Fahnøe, U.; Feng, S.; Scheel, T.K.H.; et al. Hepatitis C Virus Protease Inhibitors Show Differential Efficacy and Interactions with Remdesivir for Treatment of SARS-CoV-2 in Vitro. *Antimicrob. Agents Chemother.* **2021**, *65*, e0268020.

4. Pham, L. V.; Jensen, S.B.; Fahnøe, U.; Pedersen, M.S.; Tang, Q.; Ghanem, L.; Ramirez, S.; Humes, D.; Serre, S.B.N.; Schønning, K.; et al. HCV Genotype 1-6 NS3 Residue 80 Substitutions Impact Protease Inhibitor Activity and Promote Viral Escape. *J. Hepatol.* **2019**, *70*, 388–397.

5. Fahnøe, U.; Bukh, J. Full-Length Open Reading Frame Amplification of Hepatitis C Virus. *Methods Mol. Biol.* **2019**, *1911*, 85–91.

6. Zhou, Y.; Gilmore, K.; Ramirez, S.; Settles, E.; Gammeltoft, K.A.; Pham, L. V; Fahnøe, U.; Feng, S.; Offersgaard, A.; Trimpert, J.; et al. In Vitro Efficacy of Artemisinin-Based Treatments against SARS-CoV-2. *Sci Rep* **2021**, *11*, 15471.

7. Chou, T.C.; Talalay, P. Quantitative Analysis of Dose-Effect Relationships: The Combined Effects of Multiple Drugs or Enzyme Inhibitors. *Adv. Enzyme Regul.* **1984**, *22*, 27–55.

8. Chou, T.-C. and Martin, N. CompuSyn Software for Drug Combinations and for General Doseeffect Analysis, and User's Guide. ComboSyn, Inc. Paramus, NJ [Www.Combosyn.Com] 2007.
